# Sustained efficacy of adjuvant immunotherapy with cytokine-induced killer cells for hepatocellular carcinoma: an extended 5-year follow-up

**DOI:** 10.1007/s00262-018-2247-4

**Published:** 2018-09-19

**Authors:** Jeong-Hoon Lee, Joon Hyeok Lee, Young-Suk Lim, Jong Eun Yeon, Tae-Jin Song, Su Jong Yu, Geum-Youn Gwak, Kang Mo Kim, Yoon Jun Kim, Jae Won Lee, Jung-Hwan Yoon

**Affiliations:** 1 Department of Internal Medicine and Liver Research Institute, Seoul National University College of Medicine, 103 Daehak-ro, Jongno-gu, Seoul, 03080 South Korea; 2 Department of Medicine, Samsung Medical Center, Sungkyunkwan University School of Medicine, Seoul, South Korea; 3 Department of Gastroenterology, Asan Medical Center, University of Ulsan College of Medicine, Seoul, South Korea; 4 The Liver Center, Korea University Guro Hospital, Seoul, South Korea; 5 Department of Surgery, Korea University Ansan Hospital, Ansan, Gyeonggi-do South Korea; 6 Department of Statistics, Korea University, Seoul, South Korea

**Keywords:** Hepatocellular carcinoma, Cytokine-induced killer cell, Adjuvant immunotherapy, Recurrence-free survival, Overall survival

## Abstract

Our earlier multicenter randomized controlled trial showed that adjuvant immunotherapy with cytokine-induced killer (CIK) cells resulted in longer recurrence-free survival (RFS) and overall survival (OS) as well in patients who received curative treatment for hepatocellular carcinoma (HCC). In the present study, we determined if the efficacy of CIK cell therapy continued after end of repeated CIK cell injections. We performed a follow-up study of our preceding trial. We included 226 patients: 114 patients in the immunotherapy group (injection of 6.4 × 10^9^ CIK cells, 16 times during 60 weeks) and 112 patients in the control group (no treatment) after potentially curative treatment for HCC. In total, 162 patients (89 of the immunotherapy group and 73 of controls) underwent an extended follow-up for 60 months after randomization of the last patient. The primary endpoint was RFS, and secondary endpoints included OS. During follow-up time of median 68.5 months (interquartile range 45.0–82.2 months), the immunotherapy group continued to show a significantly lower risk of recurrence or death [hazard ratio (HR) 0.67; 95% confidence interval (CI) 0.48–0.94; *P* = 0.009 by one-sided log-rank test]. At 5 years, RFS rate was 44.8% in the immunotherapy group and 33.1% in the control group. The risk of all-cause death was also lower in the immunotherapy group compared to the control group (HR 0.33; 95% CI 0.15–0.76; *P* = 0.006). In patients who received curative treatment for HCC, the significant improvement in RFS and OS as a result of adjuvant CIK cell immunotherapy lasted over 5 years without boosting.

**Electronic supplementary material:**

The online version of this article (10.1007/s00262-018-2247-4) contains supplementary material, which is available to authorized users.

## Introduction

Liver cancer is the sixth most frequent cancer and the second leading cause of cancer-related mortality in the world [3]. The nationwide establishment of hepatocellular carcinoma (HCC) surveillance programs among high-risk subjects has increased the possibility of curative treatment [4, 5]. However, even after curative resection of HCC, the risk of recurrence is reportedly very high, up to 70% after 5 years. Furthermore, the median time to recurrence is < 3 years [6, 7], indicating that poor prognosis remains an issue. To date, the benefits of adjuvant therapy are still uncertain [8, 9], and current international practice guidelines do not advocate any adjuvant therapy after potentially curative treatment [10, 11].

Recently, our group published the results of a multicenter, open-labeled, randomized controlled trial (RCT) demonstrating that adjuvant adoptive immunotherapy using autologous cytokine-induced killer (CIK) cells prolongs both recurrence-free survival (RFS) and overall survival (OS) after potentially curative treatment for HCC [2]. CIK cells are polyclonal T lymphocytes, which are expanded ex vivo with cytokines and include CD3^−^/CD56^+^ natural killer (NK) cells, CD3^+^/CD56^+^ NK/T-like cells, and CD3^+^/CD56^−^ cytotoxic T cells. CD3^+^/CD56^+^ cells are scare in fresh human peripheral blood, and they are the main antitumor effector cells [12]. These cells grow rapidly and have strong antitumor effects with the capability of both T cells and NK cells, and they display minimal cytotoxicity to normal cells but substantial specific cytotoxicity for tumor cells [13, 14]. Patients who underwent adjuvant CIK cell immunotherapy (injection of autologous CIK cell agent 16 times during 60 weeks) had a significantly longer RFS compared to control patients (median 44.0 vs. 30.0 months) with 37% lower risk of recurrence or death [hazard ratio (HR) 0.63; 95% confidence interval (CI) 0.43–0.94]. CIK cell immunotherapy was also linked to a reduced risk of both all-cause death (HR 0.21; 95% CI 0.06–0.75) and cancer-related death (HR 0.19; 95% CI 0.04–0.87) [2].

In the original study, adjuvant CIK cell therapy improved RFS by decreasing early recurrence (within the first 2 years) but not late recurrence (beyond 2 years) during the median follow-up of approximately 3 years (40.0 months and 36.5 months in the immunotherapy group and control group, respectively), which was consistent with previous studies [15–17]. Thus, an extended follow-up study was required to determine whether the effects of CIK cell immunotherapy as an adjuvant treatment persist over a long-term period.

In this study, we assessed the long-term effectiveness of a CIK cell agent in an adjuvant therapy for HCC by extending the follow-up time of the original RCT.

## Patients and methods

### Study design

We previously reported the findings of a 2-year follow-up after enrollment of the last patient. In that study, 230 patients received potentially curative treatment [surgical resection, radiofrequency ablation (RFA), or percutaneous ethanol injection (PEI)] for HCC. Patients with pre-treatment clinical stage I or II HCC according to the American Joint Committee on Cancer (AJCC) staging system (6th edition) [18] and based on radiological imaging studies, were recruited at five university-affiliated hospitals in Korea. HCC was diagnosed by pathological evaluation or radiological imaging studies [19]. Eligibility criteria also included hepatic function of Child-Pugh class A and an Eastern Cooperative Oncology Group performance status score of 0 or 1. Exclusion criteria included autoimmune disease or immunodeficiency, previous or current malignant tumor other than HCC, and severe allergic disorders. Pregnant or breastfeeding women and women planning to become pregnant were also excluded. All eligible participants were randomly assigned to receive adjuvant immunotherapy using a CIK cell agent (the immunotherapy group) or no adjuvant treatment (control group) in a 1:1 ratio. Subjects in the immunotherapy group received the CIK cell agent (Immuncell-LC; GREEN CROSS CELL Corp., Seoul, Korea) intravenously 16 times during 60 weeks (4 treatments once a week, followed by 4 treatments every other week, then 4 treatments every 4 weeks, and finally 4 treatments every 8 weeks) after curative treatment for HCC. The CIK cell agent was manufactured at a central facility. Briefly, mononuclear cells were isolated from 120 mL of autologous peripheral blood from each patient and externally cultured at 37 °C for 12–21 days in the presence of interleukin-2 and immobilized monoclonal antibody to CD3, following a modified method [13, 20]. The mean cell count in the CIK cell agent was 6.4 × 10^9^ cells. Of the 230 randomized patients, 226 (114 and 112 in the immunotherapy group and the control group, respectively) were included in the efficacy analysis: following a decision from the steering committee, 4 patients were excluded from the efficacy population because 1 in the immunotherapy group and 3 in the control group were found to have violated the inclusion or exclusion criteria. The CIK cell agent was administered 13.8 times (mean) in the immunotherapy group.

Among the 226 patients recruited in the efficacy population of the original study, patients who died or withdrew consent during the study period, or were lost to follow-up were not eligible for the extended follow-up study. Patients who declined to extend the follow-up were also excluded. Finally, 162 patients (71.7%; 89 in the immunotherapy group and 73 in the control group) agreed to be included in the extended follow-up and provided written informed consent. They were followed up for 60 months after randomization of the last patient. For the remaining 64 patients, the data from the original study were used for statistical analyses.

### Endpoints and assessments

The primary endpoint was RFS, which was measured from the date of randomization to the first recurrence or death from any cause. The secondary endpoints included OS and cancer-specific survival (CSS). OS was defined as the time between randomization and death due to any cause, and CSS was defined as time between randomization and death due to HCC.

Tumors were evaluated by contrast-enhanced computed tomography or magnetic resonance imaging every 3 months for 24 months and then every 3–6 months. Two independent radiologists with > 5 years of experience and blinded to the group assignment reviewed all scans at each site. In cases of disagreement, a third radiologist independently reviewed images, and the case was decided by consensus. The data cutoff date for extended follow-up was January 29, 2016. For patients who did not agree to participate in the extended follow-up study, the data cutoff date was November 29, 2012, as in the original study.

### Statistical analyses

Efficacy outcomes were assessed using an intention-to-treat approach. Kaplan–Meier curves were constructed for RFS, OS, and CSS. Cox proportional hazards analysis was used to estimate unadjusted HRs. The consistency of the treatment effects on the primary endpoint with immunotherapy compared with no immunotherapy was evaluated by pre-specified subgroup analyses. In addition, we performed a post hoc analysis to determine whether the risk of tumor recurrence or death was comparable between groups after immunotherapy with a finite duration. Patients in the immunotherapy group who did not experience tumor recurrence or death during treatment and all patients in the control group were included in the post hoc analysis, in which index dates were defined as the last date of CIK cell injection in the immunotherapy group and the date of randomization in the control group. The Cox proportional hazards regression model was used to evaluate the effect of baseline characteristics on each endpoint. The log-rank test for the primary endpoint was one-sided, and all other statistical tests were two-sided. Statistical significance was set at *P* < 0.05. Statistical analyses were performed by independent statisticians using SAS software version 9.2 (SAS Institute Inc., Cary, NC), R package version 2.15.3 (R Foundation for Statistical Computing, Vienna, Austria), and Stata software version 13.0 (StataCorp, College Station, TX).

## Results

### Patients

**Fig. 1:**
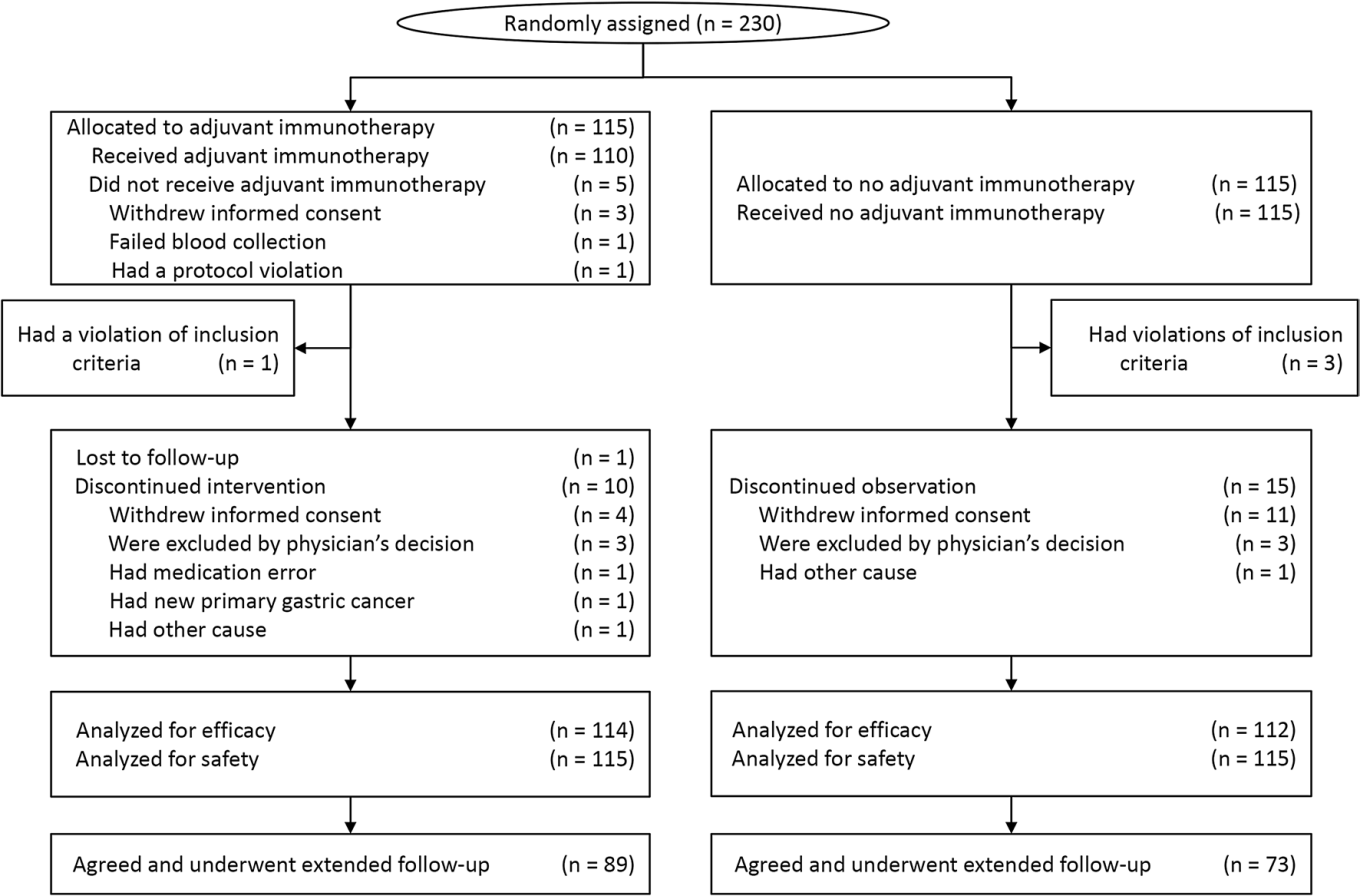
CONSORT diagram. Among the patients who were enrolled in the original study, 89 patients in the immunotherapy group and 73 patients in the control group agreed to participate and completed the extended follow-up

The efficacy of CIK cell immunotherapy was analyzed for all 226 patients included in the original study. Of these patients, 64 were included in the extended follow-up: 17 patients died and 15 withdrew their consent during the original study period, 10 were lost to follow-up, and 22 declined to participate in the extended follow-up. Baseline characteristics of patients who were not part of the extended follow-up did not differ significantly between the immunotherapy and control groups (Supplementary Table 1). Finally, 162 patients (71.7%; 89 in the immunotherapy group and 73 in the control group) agreed to participate in the extended follow-up and signed an informed consent form. The trial profiles of the participants in the extended follow-up study are shown in Fig. 1.

At the time of data cutoff, the overall median follow-up duration was 68.5 months [interquartile range (IQR) 45.0–82.0 months]: 70.5 months (IQR 62.0–83.0 months) in the immunotherapy group and 67.0 months (IQR 37.0–81.0 months) in the control group. Baseline characteristics of the efficacy population are provided in Table 1. Maximal tumor size was significantly larger and platelet count was lower in the immunotherapy group compared to control, as in the original study. Chronic hepatitis B virus (HBV) infection was the most common underlying liver disease in patients and about 65% had clinical or radiological evidence of liver cirrhosis. The time interval and modality of imaging studies were comparable between the two study groups (Supplementary Table 2).

**Table 1:** Baseline demographics and disease characteristics

Variables	Immunotherapy (*n* = 114)	Control (*n* = 112)	*P* value
Male sex, *N* (%)	95 (83.3%)	91 (81.3%)	0.68^e^
Age, years	55.4 ± 8.2	56.4 ± 10.6	0.41^*f*^
Treatment modality, *N* (%)			0.06^g^
PEI	13 (11.4%)	4 (3.6%)	
RFA	69 (60.5%)	70 (62.5%)	
Surgical resection	32 (28.1%)	38^d^ (33.9%)	
HCC stage, *N* (%)^a^			0.67^e^
Stage I	98 (86.0%)	94 (83.9%)	
Stage II	16 (14.0%)	18 (16.1%)	
Number of HCC, *N* (%)			0.98^e^
< 3	112 (98.2%)	110 (98.2%)	
≥ 3	2 (1.8%)	2 (1.8%)	
Size of HCC, cm	1.8 (1.4–2.3)	2.3 (1.5–3.1)	0.03^h^
ECOG status, *N* (%)^b^			0.83^e^
0	81 (71.1%)	81 (72.3%)	
1	33 (28.9%)	31 (27.7%)	
Underlying liver disease, *N* (%)			0.87^g^
HBV infection only	96 (84.2%)	90 (80.4%)	
HCV infection only	9 (7.9%)	10 (8.9%)	
HBV + HCV co-infection	2 (1.8%)	2 (1.8%)	
Others	7 (6.1%)	10 (8.9%)	
Cirrhosis, *N* (%)^c^	76 (66.7%)	70 (62.5%)	0.51^e^
Alpha-fetoprotein, ng/mL	5.2 (3.1–9.9)	5.4 (3.0–13.0)	0.56^h^
PIVKA-II, mAU/mL	19.0 (14.0–24.8)	18.0 (14.0–24.0)	0.96^h^
AST, IU/L	33.0 (27.0–43.5)	34.0 (26.8–44.0)	0.87^h^
ALT, IU/L	33.0 (25.0–45.8)	33.0 (23.0–47.5)	0.55^h^
ALP, IU/L	82.5 (70.0–101.5)	82.0 (65.0–100.0)	0.45^h^
Albumin, g/dL	4.1 (3.9–4.3)	4.1 (3.9–4.3)	0.99^h^
Total bilirubin, mg/dL	0.8 (0.6–1.0)	0.8 (0.6–1.0)	0.71^h^
Prothrombin time, s	13.7 (13.1–14.7)	13.9 (13.2–14.4)	0.74^h^
Creatinine, mg/dL	0.9 (0.8–1.0)	0.9 (0.7–1.0)	0.86^h^
Platelet, ×10^3^/mm^3^	116.5 (92.3–158.0)	141.0 (117.5–166.3)	0.01^h^

Data are expressed as *N* (%), mean ± SE, or median (interquartile range [Q1–Q3])

*NS* not significant, *RFA* radiofrequency ablation, *PEI* percutaneous ethanol injection, *HCC* hepatocellular carcinoma, *IQR* interquartile range, *ECOG* Eastern Cooperative Oncology Group, *HBV* hepatitis B virus, *HCV* hepatitis C virus, *PIVKA-II* protein induced by vitamin K absence-II, *AST* aspartate aminotransferase, *ALT* alanine aminotransferase, *ALP* alkaline phosphatase

^a^The HCC staging was done according to AJCC staging system (6th edition) [18]

^b^The ECOG performance status assesses the daily living abilities of the patient, on a scale ranging from 0 (fully active) to 5 (dead)

^c^Liver cirrhosis was diagnosed by the presence of histological and/or radiological evidence

^d^Two of them underwent intrahepatic RFA in addition to surgical resection

^e^By Chi-square test

^f^By two sample *t* test

^g^By Fisher’s exact test

^h^By Wilcoxon rank sum test

### Recurrence-free survival

**Fig. 2:**
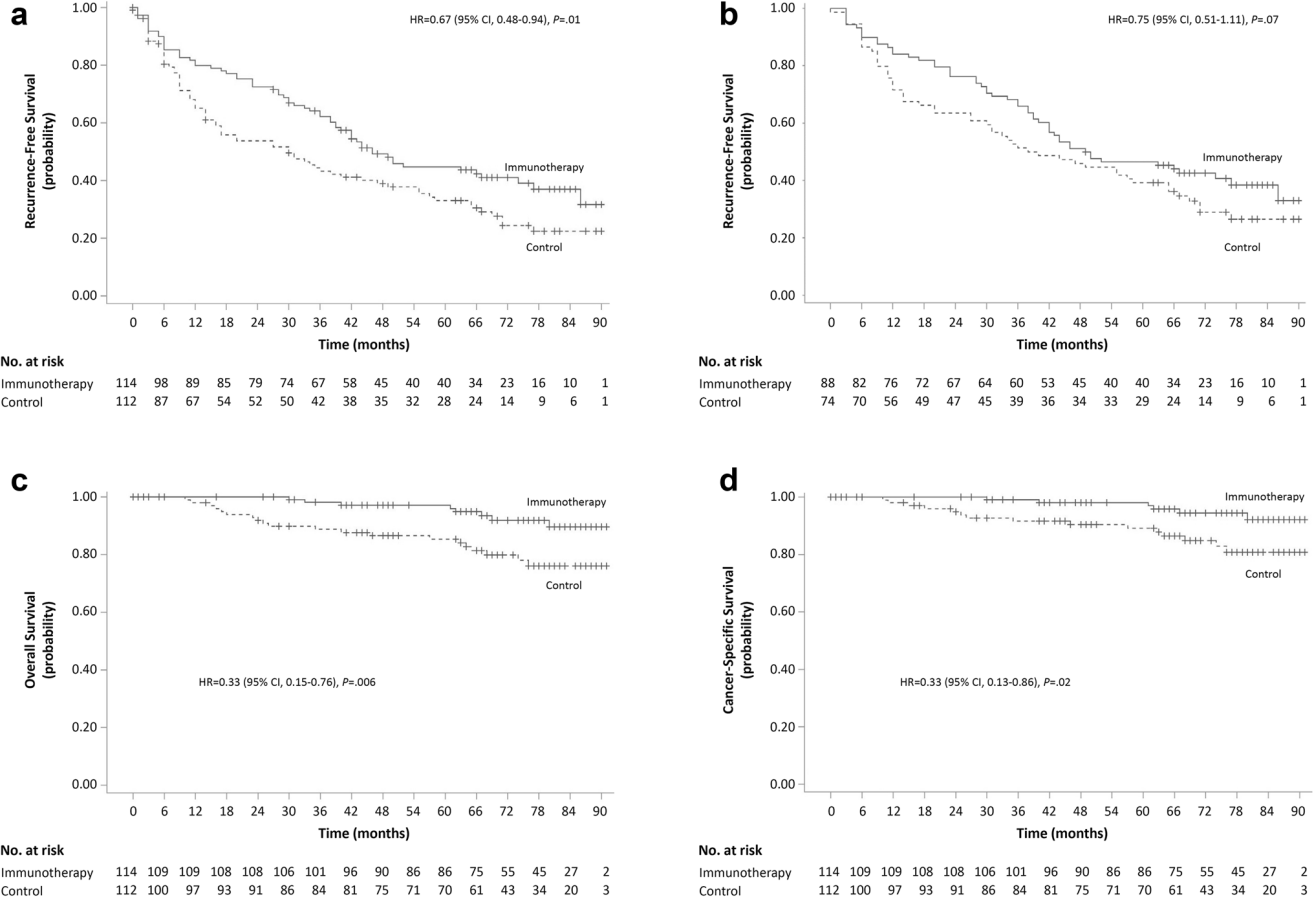
Kaplan–Meier estimates of recurrence-free survival (RFS), overall survival (OS), and cancer-specific survival (CSS). **a** RFS for overall efficacy population. **b** RFS of patients who completed the extended follow-up. **c** OS for overall efficacy population. **d** CSS for overall efficacy population

Because the median RFS was attained within the time limit of the original study, it was 14.0 months longer in the immunotherapy group (44.0 months) than in the control group (30.0 months), as in the original study. During the extended follow-up period, 44 more patients experienced tumor recurrence or death by the time of data cutoff: 21 of 89 patients (23.6%) in the immunotherapy group (16 recurrences and 5 deaths) and 23 of 73 patients (31.5%) in the control group (15 recurrences and 8 deaths). Collectively, during the entire follow-up period, 67 of 114 patients (58.8%) in the immunotherapy group (61 recurrences and 6 deaths without recurrence) and 78 of 112 patients (69.6%) in the control group (68 recurrences and 10 deaths without recurrence) experienced tumor recurrence or death. After including the extended follow-up period, the difference in RFS between the two groups remained statistically significant (*P* = 0.01 based on one-sided log-rank test). The HR with immunotherapy was 0.67 (95% CI 0.48–0.94), representing a 33% relative reduction in the immunotherapy group (Fig. 2a and Supplementary Table 3), which was comparable to the original study (0.63; 95% CI 0.43–0.94). RFS rates at 24, 36, 60, 72, and 84 months were significantly higher in the immunotherapy group than in the control group (all *P* < 0.05 based on one-sided *z* test). The 5-year RFS rate was 44.8% in the immunotherapy group and 33.1% in the control group (Supplementary Table 3).

In multivariable Cox regression using stepwise forward selection, adjuvant immunotherapy was an independent prognostic factor (adjusted HR 0.69; 95% CI 0.49–0.97; *P* = 0.016) after adjustment for age, serum alpha-fetoprotein (AFP) level, and treatment modality (Supplementary Table 4).

In a subgroup of 162 patients who agreed to participate in the extended follow-up, there was an association between CIK cell immunotherapy and longer RFS (HR 0.75; 95% CI 0.51–1.11; *P* = 0.07 based on one-sided log-rank test; Fig. 2b), but it was not statistically significant.

In a subgroup of 186 patients with HBV-related HCC (96 in the immunotherapy group and 90 in the control group), CIK cell immunotherapy was associated with significantly longer RFS (HR 0.66; 95% CI 0.46–0.96; *P* = 0.01 based on one-sided log-rank test). However, in subgroups of patients with hepatitis C virus (HCV)-related HCC (*n* = 23; HR 1.002; 95% CI 0.36–2.78; *P* = 0.50) and non-HBV/non-HCV HCC (*n* = 17; HR 0.62; 95% CI 0.17–2.25; *P* = 0.23), RFS did not differ significantly between the immunotherapy and control groups, although the HRs were similar as in the HBV-related HCC subgroup.

Regarding maximal tumor size, the HRs in the immunotherapy group were similar between patients with maximal size < 2 cm (0.71; 95% CI 0.43–1.17; *P* = 0.09 by one-sided log-rank test; *n* = 108; Supplementary Fig. 1A) and those with ≥ 2 cm (0.66; 95% CI 0.41–1.05; *P* = 0.035 based on one-sided log-rank test; *n* = 96; Supplementary Fig. 1B). HRs were similar between patients with AJCC stage I HCC (0.66; 95% CI 0.46–0.96; *P* = 0.01 based on one-sided log-rank test; *n* = 192; Supplementary Fig. 2A) and those with stage II HCC (0.82; 95% CI 0.37–1.83; *P* = 0.31 based on one-sided log-rank test; *n* = 34; Supplementary Fig. 2B). When the immunotherapy group was divided according to a cutoff value of 97.8 × 10^9^ cells, which was the median number of total injected CIK cells, there was no significant difference in RFS (≥ 97.8 × 10^9^ vs. <97.8 × 10^9^ cells HR 0.98; 95% CI 0.76–1.26; *P* = 0.87; Supplementary Fig. 3A). In addition, in a subgroup of patients in the immunotherapy group who received all scheduled 16 injections (*n* = 83), RFS did not differ between patients who received ≥ 102 × 10^9^ cells (median value of total injected CIK cells) and those who received < 102 × 10^9^ cells (HR 0.75; 95% CI 0.40–1.41; *P* = 0.37; Supplementary Fig. 3B).

In *post hoc* analysis, patients who did not have tumor recurrence or die during adjuvant immunotherapy in the immunotherapy group (*n* = 95) maintained a significantly lower risk of recurrence or death than the patients in the control group (*n* = 112), even after the cessation of immunotherapy (HR 0.71; 95% CI 0.49–1.01; *P* = 0.03 based on one-sided log-rank test; Supplementary Fig. 4).

The 131 patients (100 in the original study and additional 31 during the extended follow-up period; 63 in the immunotherapy group and 68 in the control group) who had tumor recurrence were further treated for a median of four times (IQR, 0–30) with multidisciplinary modalities such as transarterial chemoembolization, RFA, PEI, surgical resection, liver transplantation, sorafenib administration, conventional cytotoxic chemotherapy, external beam radiotherapy, and proton therapy (Supplementary Table 5).

### Overall and cancer-specific survival

During the extended follow-up period, 13 additional patients (5 in the immunotherapy group and 8 in the control group) died. Ten deaths were cancer-related (4 in the immunotherapy group and 6 in the control group), and three deaths were not HCC-related (1 in the immunotherapy group and 2 in the control group). In the immunotherapy group, a patient died of pneumonia. In the control group, one patient died of laryngeal cancer and another patient died of unknown cause. Therefore, during the entire study period, a total of 28 deaths occurred in the efficacy population: 8 patients in the immunotherapy group (6 cancer-related and 2 cancer-unrelated deaths) and 20 in the control group (15 cancer-related and 5 cancer-unrelated deaths).

Both median OS and CSS were still not attained in either group. OS was significantly longer in the immunotherapy group than in the control group (HR 0.33; 95% CI 0.15–0.76; *P* = 0.006; Fig. 2c and Supplementary Table 3). Recurrence status was significantly related to relative risk of death (3.34; 95% CI 1.32–8.46; *P* = 0.0006 based on *z* test). When the immunotherapy group was divided based on the total count of injected CIK cells, no significant difference in OS was observed (≥ 97.8 × 10^9^ vs. <97.8 × 10^9^ cells; HR 0.94; 95% CI 0.49–1.76; *P* = 0.84; Supplementary Fig. 3C). In addition, in a subgroup of patients in the immunotherapy group who received all scheduled 16 injections (*n* = 83), there was no difference in OS between patients who received ≥ 102 × 10^9^ cells (median total injected CIK cells) and those who received < 102 × 10^9^ cells (HR 0.80; 95% CI 0.13–4.76; *P* = 0.81; Supplementary Fig. 3D).

In addition, CSS was significantly longer in the immunotherapy group (HR 0.33; 95% CI 0.13–0.86; *P* = 0.02; Fig. 2d and Supplementary Table 3).

### Change in serum AFP levels

At the end of the study, mean serum AFP levels were 16.1 ± 40.5 ng/mL in the immunotherapy group and 54.0 ± 439.3 ng/mL in the control group. However, the change from baseline was not statistically significant in the two groups (5.3 ± 42.0 vs. 50.3 ± 463.2 ng/mL; *P* = 0.57; Supplementary Table 6).

## Discussion

In this extended follow-up study, patients who received adjuvant autologous CIK cell immunotherapy after potentially curative treatment for HCC maintained a significantly longer RFS, OS, and CSS than did patients who received no adjuvant immunotherapy. HRs with CIK cell immunotherapy were similar across tumor size, tumor stage, and the etiologies of underlying liver disease. The difference in RFS between the groups remained statistically significant, even after the end of adoptive immunotherapy.

The results of this study showed that the effect of adjuvant immunotherapy with CIK cells in patients who underwent curative treatment for HCC was sustained during the 6-year follow-up. Considering that the mean treatment duration of CIK cell immunotherapy was only 12.0 months and that the half-life of adoptive CIK cells is short, CIK cell immunotherapy may have additional effects beyond the direct killing of residual tumor cells. The prolonged antitumor effect of CIK cell treatment can be explained by several possible mechanisms. First, CD3^−^/CD56^+^ NK cells that are included in CIK cell agents can preferentially kill cancer stem cells [21, 22]. Cancer stem cells are a small proportion of cancer cells with the ability to maintain long-term growth potential and are responsible for tumor recurrence [23]. In addition, cancer stem cells are believed to account for the recurrence of HCC [24]. Second, some preparations of CIK cells reportedly have immune memory that could last a long time, and these cell may be later activated to kill recurrent tumor cells. CIK cell agents may induce long-lasting memory T cells. CD3^+^/CD56^+^ cells contribute to the formation and proliferation of memory CD8^+^ T cells [25]. Recent studies indicated that CD3^−^/CD56^+^ NK cell activation could lead to the generation of memory NK cells, a feature previously ascribed only to B and T cells [26, 27]. In addition, CIK cells exhibit the phenotype of terminally differentiated memory T cells with CD45RA^+^, CCR7^−^, CD62L^weak+^, CD11a^+^, CD27^+^, CD28^−^, macrophage inflammatory protein 1a^+^, perforin^+^, and Fas ligand^+^ [28]. A further study is warranted to evaluate the immune cell status immediately before and after adoptive transfer of CIK cells as well as gradual changes in the immune cell population over a long-term period.

Interestingly, in the original study, CIK cell immunotherapy reduced the risk of tumor recurrence at all types of locations, including local intrahepatic, distant intrahepatic, and extrahepatic recurrences. On the other hand, CIK cell immunotherapy failed to reduce late recurrence (beyond 2 years) although it reduced early recurrence (within 2 years) on the basis of visual evaluation of Kaplan–Meier curves [2]. Because both distant intrahepatic recurrence and late recurrence are related to de novo tumor development rather than metastasis of residual tumor cells [29], the results were conflicting. The current extended study showed that CIK cell immunotherapy significantly prolonged RFS during the off-treatment period as well as during the on-treatment period and consequently reduced late recurrence. This difference from the original study might be related to the extended follow-up duration of the current study (median 70.5 months in the immunotherapy group); the study duration of the original study (median 40.0 months in the immunotherapy group) might have been too short to evaluate the risk of late recurrence. Collectively, CIK cells can apparently reduce de novo carcinogenesis as well as clear residual tumor cells. As mentioned previously, memory T cells or NK cells induced by CIK cell treatment cleared not only residual tumor cells but also new cancer cells that develop in diseased liver, although the gap between CIK cell treatment and the development of new malignant cells is lengthy. Moreover, CD3^−^/CD56^+^ NK cells are known to preferentially kill cancer stem cells [30].

In most other cancers, adjuvant therapy is generally indicated after the resection of locally advanced tumors but not during the early stages of the disease. Adjuvant systemic treatment is recommended for stage IB or IIA gastric cancer, stage II or III colon cancer, and stage II or IIIA non-small cell lung cancer, but not for most stage I or IA cancers because the risk of tumor recurrence is low after curative resection. On the contrary, it is recommended that patients who receive curative treatment for early stage HCC be entered into clinical trials of adjuvant treatment [31]. The high chance of cancer recurrence, even after potentially curative treatment, may account for this unique recommendation. The 5-year RFS rates after potentially curative treatment were reported to be 29.1% for very early or early HCC [32] and 97.8% for early gastric cancer [32, 33]. This substantial difference results in different 5-year survival rates: 76.1% for very early or early HCC *vs*. >90% for early gastric cancer [32, 33]. Thus, there is an urgent need for an effective adjuvant therapy to improve the prognosis of HCC patients.

As an adjuvant therapy for HCC after curative treatment, CIK cells have been widely evaluated in Asian countries including Korea, Japan, and China. Recently, another RCT also showed that CIK cell treatment significantly increased time-to-recurrence after curative resection [34]. A recent meta-analysis involving 12 RCTs evaluating the efficacy of adjuvant autologous CIK cell treatment showed that CIK cell treatment significantly prolonged both RFS (pooled HR 0.56; 95% CI 0.47–0.67) and OS (HR 0.59; 95% CI 0.46–0.77) although heterogeneity was moderate (both *I*^2^ = 48%) [35]. Among eight RCTs that evaluated RFS as an endpoint, seven trials demonstrated that CIK cell treatment significantly improved RFS with HRs ranging from 0.14 to 0.63 [2, 15–17, 36, 37]. Although the number of CIK cell injections varied from 3 to 16, the studies consistently showed significant improvement in RFS. OS was significantly prolonged by CIK cell treatment in five trials [2, 38–40]. To date, adoptive CIK cell immunotherapy has been the only adjuvant therapy proven to improve both RFS and OS. Sorafenib, a multitargeted tyrosine kinase inhibitor, failed to improve RFS in a recent large-scale RCT involving 1,114 patients [41].

Adoptive immunotherapy using autologous CIK cells has several limitations. The effectiveness of ex vivo CIK cell expansion varies greatly among patients according to the extent of the patient’s immunosuppression. Myeloid-derived suppressor cells and defective antigen-presenting cells can inhibit CIK cell expansion. The quantity and quality of T lymphocytes are poor in cancer patients [42]. Therefore, the efficacy of CIK cell therapy should be improved using several possible methods. First, combination therapy with immune checkpoint inhibitors, such as inhibitors of programmed death-1, programmed death-ligand 1, and cytotoxic T-lymphocyte-associated protein 4, could be used to inhibit immune evasion by malignant cells and to enhance antitumor activity of CIK cells [43, 44]. Second, CIK cells could be stimulated by upregulating the expression of major histocompatibility complex (MHC) class I-related chain (MIC)-A and -B, which bind to natural killer group 2 member D (NKG2D) and activate CIK cells. Our group recently reported that histone deacetylase inhibitors such as suberoylanilide hydroxamic acid and valproate enhance the expression of MIC-A and -B in an epigenetic manner [45]; therefore, combination therapy with these agents could also be considered. Third, combination treatment with different types of adoptive immunotherapy such as dendritic cell vaccine could be utilized. CIK cells also include a number of cytotoxic T lymphocytes that have an MHC-restricted cytotoxicity, which can be potentiated by high levels of MHC class I molecules and tumor antigens provided by dendritic cells. Thus, a synergistic effect of combination therapy could be expected [46]. Fourth, the downregulation of immune suppressor cells could help potentiate CIK cell immunotherapy. Low-dose cyclophosphamide was reported to suppress regulatory T-cells [47], and the inhibition of signal transducer and activator of transcription 3 (STAT3) signaling was shown to decrease levels of myeloid-derived suppressor cells [48]. Further preclinical and clinical studies are required to evaluate whether these strategies might enhance the antitumor activity of CIK cells. In addition, the long-term safety and the cost-effectiveness of adjuvant immunotherapy with CIK cells, which were not addressed in this extended follow-up study, need to be further evaluated.

## Conclusion

This extended follow-up study demonstrated that the significant increase in RFS and OS afforded by adjuvant immunotherapy with autologous CIK cells was still present for more than 5 years without boosting in patients who underwent curative treatment for HCC.

## Electronic supplementary material

Below is the link to the electronic supplementary material.

Supplementary material 1 (PDF 305 KB)

## Abbreviations

AFP: Alpha-fetoprotein
AJCC: American Joint Committee on Cancer
CI: Confidence interval
CIK: Cytokine-induced killer
CSS: Cancer-specific survival
HBV: Hepatitis B virus
HCC: Hepatocellular carcinoma
HCV: Hepatitis C virus
HR: Hazard ratio
IQR: Interquartile range
MHC: Major histocompatibility complex
MIC: Major histocompatibility complex class I-related chain
NK: Natural killer
OS: Overall survival
PEI: Percutaneous ethanol injection
RCT: Randomized controlled trial
RFA: Radiofrequency ablation
RFS: Recurrence-free survival

## References

[1] Lee JH, Lee JH, Lim YS (2018). Sustained efficacy of adjuvant immunotherapy with cytokine-induced killer cells for hepatocellular carcinoma: an extended 5-year follow-up. J Hepatol.

[2] Lee JH, Lee JH, Lim YS (2015). Adjuvant immunotherapy with autologous cytokine-induced killer cells for hepatocellular carcinoma. Gastroenterology.

[3] Ferlay J, Soerjomataram I, Ervik M et al (2013) GLOBOCAN 2012: estimated cancer incidence, mortality and prevalence Worldwide in 2012. v1.0: IARC Cancer Base No. 11. International agency for research on cancer, Lyon, France

[4] Yuen MF, Cheng CC, Lauder IJ, Lam SK, Ooi CG, Lai CL (2000). Early detection of hepatocellular carcinoma increases the chance of treatment: Hong Kong experience. Hepatology.

[5] Bolondi L, Sofia S, Siringo S (2001). Surveillance programme of cirrhotic patients for early diagnosis and treatment of hepatocellular carcinoma: a cost effectiveness analysis. Gut.

[6] Lai EC, Fan ST, Lo CM, Chu KM, Liu CL, Wong J (1995). Hepatic resection for hepatocellular carcinoma. An audit of 343 patients. Ann Surg.

[7] Shah SA, Cleary SP, Wei AC (2007). Recurrence after liver resection for hepatocellular carcinoma: risk factors, treatment, and outcomes. Surgery.

[8] Samuel M, Chow PK, Chan Shih-Yen E, Machin D, Soo KC (2009). Neoadjuvant and adjuvant therapy for surgical resection of hepatocellular carcinoma. Cochrane Database Syst Rev.

[9] Schwartz JD, Schwartz M, Mandeli J, Sung M (2002). Neoadjuvant and adjuvant therapy for resectable hepatocellular carcinoma: review of the randomised clinical trials. Lancet Oncol.

[10] Heimbach JK, Kulik LM, Finn RS, Sirlin CB, Abecassis MM, Roberts LR, Zhu AX, Murad MH, Marrero JA (2018). AASLD guidelines for the treatment of hepatocellular carcinoma. Hepatology.

[11] European Association for the Study of the Liver (2018). EASL Clinical Practice Guidelines: management of hepatocellular carcinoma. J Hepatol.

[12] Schmidt-Wolf IG, Lefterova P, Mehta BA, Fernandez LP, Huhn D, Blume KG, Weissman IL, Negrin RS (1993). Phenotypic characterization and identification of effector cells involved in tumor cell recognition of cytokine-induced killer cells. Exp Hematol.

[13] Ochoa AC, Gromo G, Alter BJ, Sondel PM, Bach FH (1987). Long-term growth of lymphokine-activated killer (LAK) cells: role of anti-CD3, beta-IL 1, interferon-gamma and -beta. J Immunol.

[14] Verneris MR, Ito M, Baker J, Arshi A, Negrin RS, Shizuru JA (2001). Engineering hematopoietic grafts: purified allogeneic hematopoietic stem cells plus expanded CD8 + NK-T cells in the treatment of lymphoma. Biol Blood Marrow Transpl.

[15] Takayama T, Sekine T, Makuuchi M (2000). Adoptive immunotherapy to lower postsurgical recurrence rates of hepatocellular carcinoma: a randomised trial. Lancet.

[16] Weng DS, Zhou J, Zhou QM (2008). Minimally invasive treatment combined with cytokine-induced killer cells therapy lower the short-term recurrence rates of hepatocellular carcinomas. J Immunother.

[17] Hui D, Qiang L, Jian W, Ti Z, Da-Lu K (2009). A randomized, controlled trial of postoperative adjuvant cytokine-induced killer cells immunotherapy after radical resection of hepatocellular carcinoma. Dig Liver Dis.

[18] Greene FL, American Joint Committee on Cancer, American Cancer Society (2002). AJCC cancer staging manual.

[19] Bruix J, Sherman M, Practice Guidelines Committee. American Association for the Study of Liver Diseases (2005). Management of hepatocellular carcinoma. Hepatology.

[20] Anderson PM, Bach FH, Ochoa AC (1988). Augmentation of cell number and LAK activity in peripheral blood mononuclear cells activated with anti-CD3 and interleukin-2. Preliminary results in children with acute lymphocytic leukemia and neuroblastoma. Cancer Immunol Immunother.

[21] Avril T, Vauleon E, Hamlat A, Saikali S, Etcheverry A, Delmas C, Diabira S, Mosser J, Quillien V (2012). Human glioblastoma stem-like cells are more sensitive to allogeneic NK and T cell-mediated killing compared with serum-cultured glioblastoma cells. Brain Pathol.

[22] Tallerico R, Todaro M, Di Franco S (2013). Human NK cells selective targeting of colon cancer-initiating cells: a role for natural cytotoxicity receptors and MHC class I molecules. J Immunol.

[23] Singh SK, Hawkins C, Clarke ID, Squire JA, Bayani J, Hide T, Henkelman RM, Cusimano MD, Dirks PB (2004). Identification of human brain tumour initiating cells. Nature.

[24] Xu XL, Xing BC, Han HB (2010). The properties of tumor-initiating cells from a hepatocellular carcinoma patient’s primary and recurrent tumor. Carcinogenesis.

[25] Eberl G, Brawand P, MacDonald HR (2000). Selective bystander proliferation of memory CD4^+^ and CD8^+^ T cells upon NK T or T cell activation. J Immunol.

[26] Cooper MA, Elliott JM, Keyel PA, Yang L, Carrero JA, Yokoyama WM (2009). Cytokine-induced memory-like natural killer cells. Proc Natl Acad Sci USA.

[27] Paust S, Gill HS, Wang BZ (2010). Critical role for the chemokine receptor CXCR6 in NK cell-mediated antigen-specific memory of haptens and viruses. Nat Immunol.

[28] Lu PH, Negrin RS (1994). A novel population of expanded human CD3^+^ CD56^+^ cells derived from T cells with potent in vivo antitumor activity in mice with severe combined immunodeficiency. J Immunol.

[29] Chen YJ, Yeh SH, Chen JT, Wu CC, Hsu MT, Tsai SF, Chen PJ, Lin CH (2000). Chromosomal changes and clonality relationship between primary and recurrent hepatocellular carcinoma. Gastroenterology.

[30] Ames E, Canter RJ, Grossenbacher SK (2015). NK cells preferentially target tumor cells with a cancer stem cell phenotype. J Immunol.

[31] Llovet JM, Di Bisceglie AM, Bruix J (2008). Design and endpoints of clinical trials in hepatocellular carcinoma. J Natl Cancer Inst.

[32] Cho EJ, Lee JH, Yoo JJ (2013). Serum insulin-like growth factor-I level is an independent predictor of recurrence and survival in early hepatocellular carcinoma: a prospective cohort study. Clin Cancer Res.

[33] Youn HG, An JY, Choi MG, Noh JH, Sohn TS, Kim S (2010). Recurrence after curative resection of early gastric cancer. Ann Surg Oncol.

[34] Xu L, Wang J, Kim Y (2016). A randomized controlled trial on patients with or without adjuvant autologous cytokine-induced killer cells after curative resection for hepatocellular carcinoma. Oncoimmunology.

[35] Cai XR, Li X, Lin JX (2017). Autologous transplantation of cytokine-induced killer cells as an adjuvant therapy for hepatocellular carcinoma in Asia: an update meta-analysis and systematic review. Oncotarget.

[36] Wang JP, Li W, Huang ZL (2012). Value of CIK in the treatment of TACE combined with RFA for HCC in long-term survival and prognostic analysis. Zhonghua Yi Xue Za Zhi.

[37] Cui J, Wang N, Zhao H, Jin H, Wang G, Niu C, Terunuma H, He H, Li W (2014). Combination of radiofrequency ablation and sequential cellular immunotherapy improves progression-free survival for patients with hepatocellular carcinoma. Int J Cancer.

[38] Hao MZ, Lin HL, Chen Q, Ye YB, Chen QZ, Chen MS (2010). Efficacy of transcatheter arterial chemoembolization combined with cytokine-induced killer cell therapy on hepatocellular carcinoma: a comparative study. Chin J Cancer.

[39] Qiu Y, Xu MB, Yun MM, Wang YZ, Zhang RM, Meng XK, Ou-Yang XH, Yun S (2011). Hepatocellular carcinoma-specific immunotherapy with synthesized alpha1,3-galactosyl epitope-pulsed dendritic cells and cytokine-induced killer cells. World J Gastroenterol.

[40] Yu X, Zhao H, Liu L (2014). A randomized phase II study of autologous cytokine-induced killer cells in treatment of hepatocellular carcinoma. J Clin Immunol.

[41] Bruix J, Takayama T, Mazzaferro V (2015). Adjuvant sorafenib for hepatocellular carcinoma after resection or ablation (STORM): a phase 3, randomised, double-blind, placebo-controlled trial. Lancet Oncol.

[42] Dugast AS, Haudebourg T, Coulon F (2008). Myeloid-derived suppressor cells accumulate in kidney allograft tolerance and specifically suppress effector T cell expansion. J Immunol.

[43] Poh SL, Linn YC (2016). Immune checkpoint inhibitors enhance cytotoxicity of cytokine-induced killer cells against human myeloid leukaemic blasts. Cancer Immunol Immunother.

[44] Zhang L, Wang J, Wei F (2016). Profiling the dynamic expression of checkpoint molecules on cytokine-induced killer cells from non-small-cell lung cancer patients. Oncotarget.

[45] Lee DH, Nam JY, Chang Y (2017). Synergistic effect of cytokine-induced killer cell with valproate inhibits growth of hepatocellular carcinoma cell in a mouse model. Cancer Biol Ther.

[46] Anguille S, Smits EL, Lion E, van Tendeloo VF, Berneman ZN (2014). Clinical use of dendritic cells for cancer therapy. Lancet Oncol.

[47] Greten TF, Ormandy LA, Fikuart A, Hochst B, Henschen S, Horning M, Manns MP, Korangy F (2010). Low-dose cyclophosphamide treatment impairs regulatory T cells and unmasks AFP-specific CD4^+^ T-cell responses in patients with advanced HCC. J Immunother.

[48] Vasquez-Dunddel D, Pan F, Zeng Q (2013). STAT3 regulates arginase-I in myeloid-derived suppressor cells from cancer patients. J Clin Invest.

