# Supplementary material for: Sustained efficacy of adjuvant immunotherapy with cytokine-induced killer cells for hepatocellular carcinoma: an extended 5-year follow-up
Source: Cancer Immunol Immunother. 2018 Sep 19;68(1):23–32. doi: 10.1007/s00262-018-2247-4 (PMC6326973; doi:10.1007/s00262-018-2247-4)
Supplement: Supplementary file 1 — Supplementary material 1 (PDF 305 KB) [file 262_2018_2247_MOESM1_ESM.pdf]

Supplement

Supplementary Figure 1. Kaplan-Meier estimates of recurrence-free survival according to maximal tumor stage. p. 2

Supplementary Figure 2. Kaplan-Meier estimates of recurrence-free survival according to AJCC stage. p. 3

Supplementary Figure 3. Kaplan-Meier estimates of recurrence-free and overall survivals according to total number of injected CIK cells. p. 4

Supplementary Figure 4. Kaplan-Meier estimates of recurrence-free survival during off-treatment period. p. 5

Supplementary Table 1. Baseline demographics and disease characteristics of patients who did not undergo the extended follow-up p. 6

Supplementary Table 2. Summary of imaging studies p. 9

Supplementary Table 3. Summary of efficacy measures (efficacy population). p. 10

Supplementary Table 4. Univariate and multivariate analyses of factors associated with recurrence-free survival. p. 12

Supplementary Table 5. Post-recurrence treatment modalities in the immunotherapy group and the control group. p. 15

Supplementary Table 6. Change in serum level of alpha-fetoprotein (efficacy population). p. 16

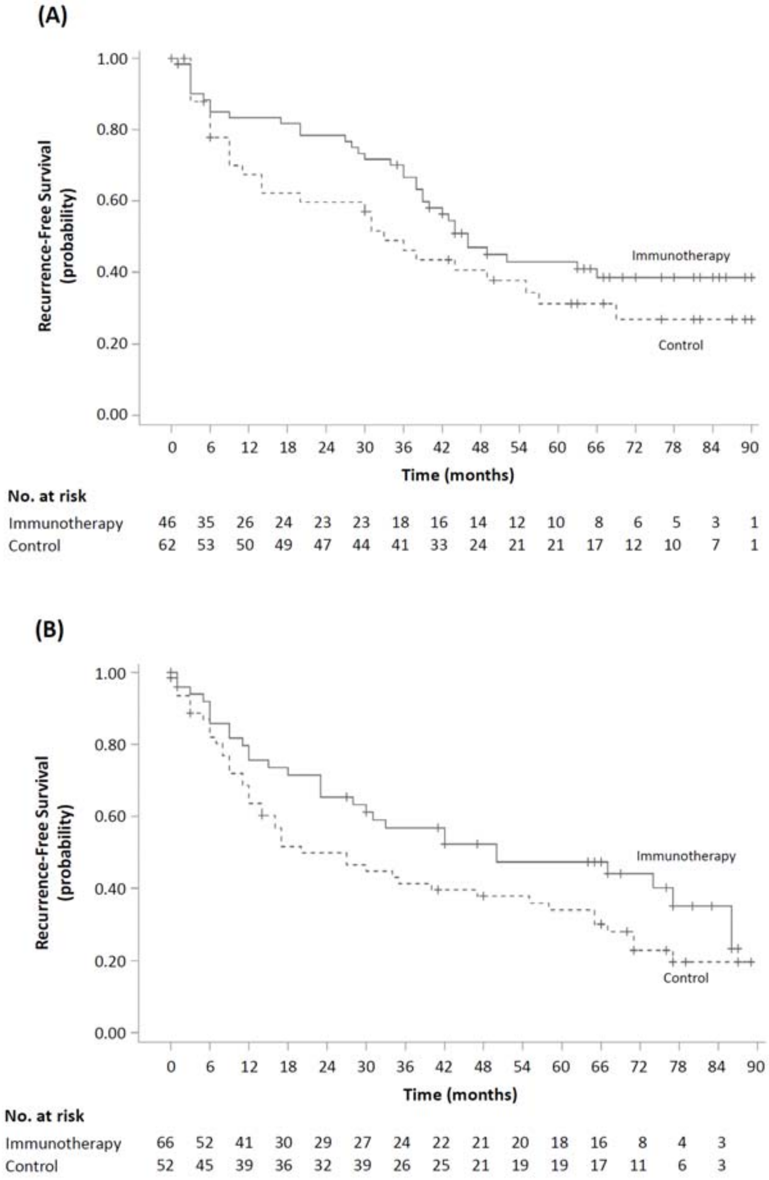

Supplementary Figure 1. Kaplan-Meier estimates of recurrence-free survival according to maximal tumor size: (A) maximal size <2 cm (HR, 0.71; 95% CI, 0.43–1.17; *P*=0.09 by one-sided log-rank test; n=108) and (B) maximal size ≥2 cm (HR, 0.66; 95% CI, 0.41–1.05; *P*=0.035 by one-sided log-rank test). RFS was computed on all patients included in the efficacy population. Patients who had not progressed or died were censored on data cut-off.

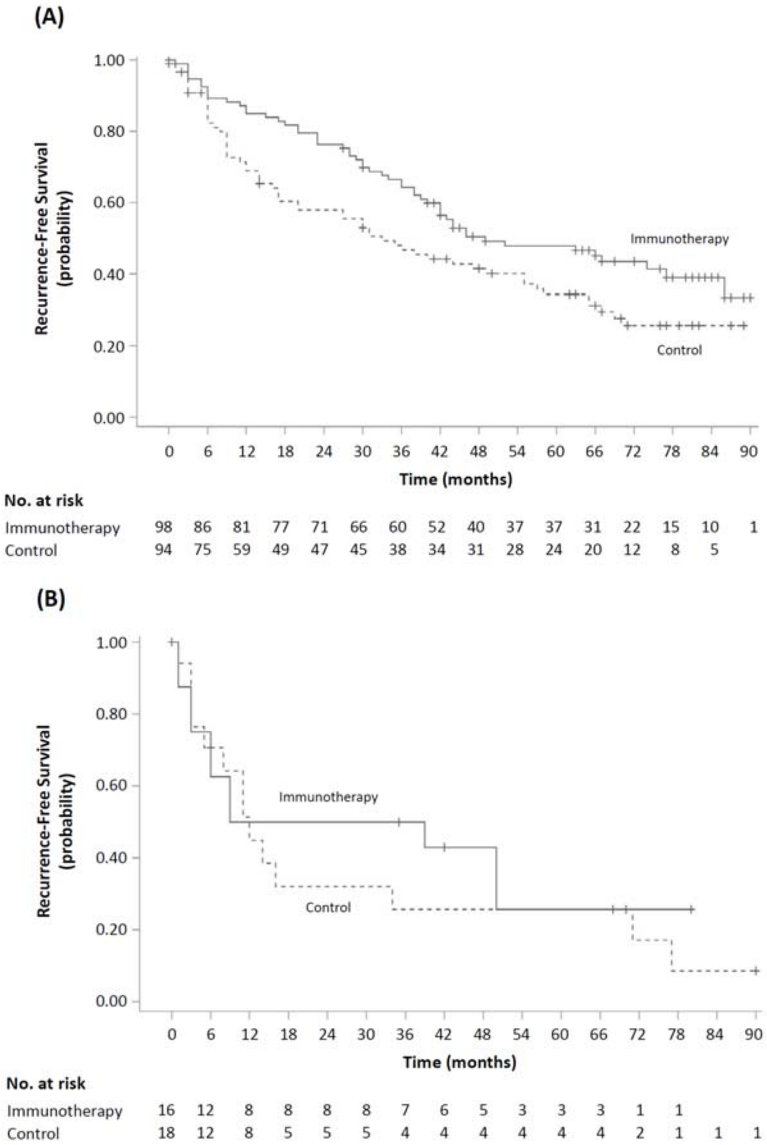

Supplementary Figure 2. Kaplan-Meier estimates of recurrence-free survival according to AJCC tumor stage: (A) stage I (HR, 0.66; 95% CI, 0.46–0.96; *P*=0.01 by one-side log-rank test) and (B) stage II (HR, 0.82; 95% CI, 0.37–1.83; *P*=0.31 by one-sided log-rank test). RFS was computed on all patients included in the efficacy population. Patients who had not progressed or died were censored on data cut-off.

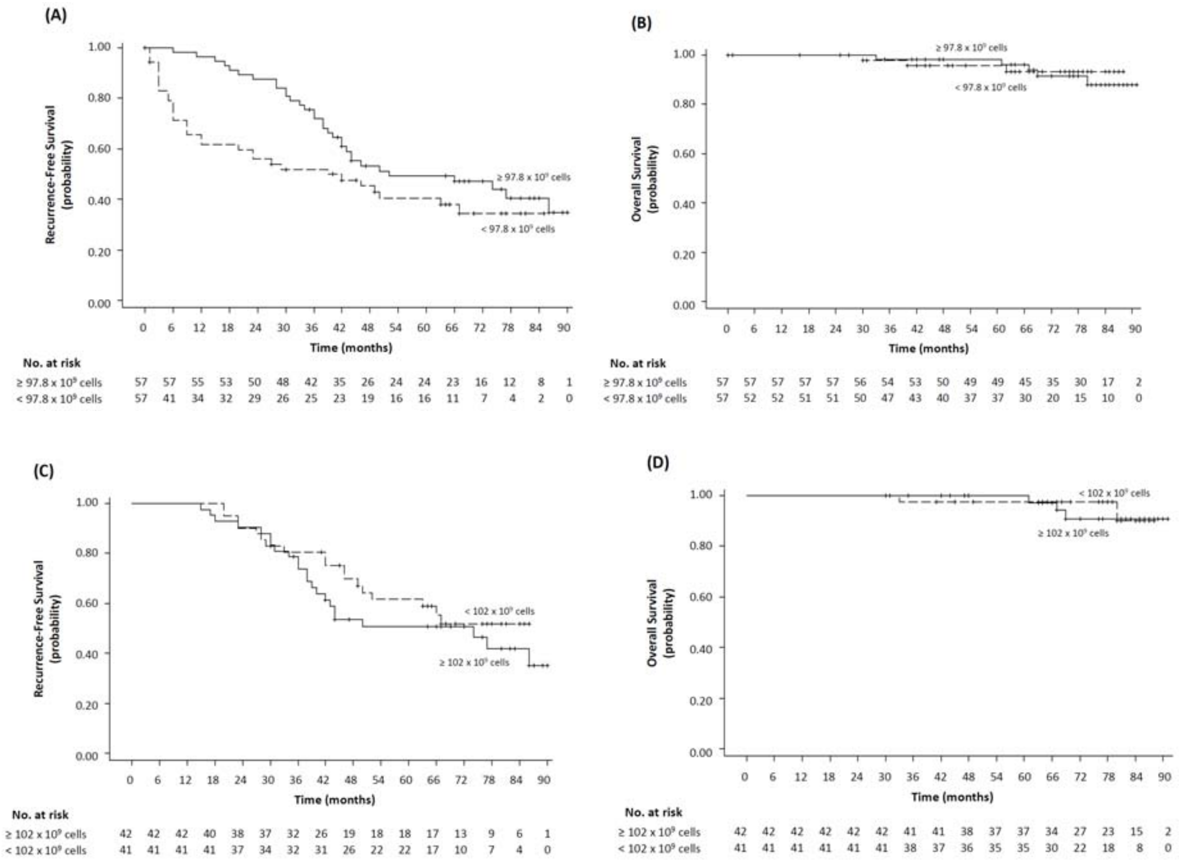

Supplementary Figure 3. Kaplan-Meier estimates of recurrence-free survival (A–B) and overall survival (C–D) according to total number of injected CIK cells: (A) among the whole immunotherapy group, ≥97.8 x 10^9 cells (median number of total injected CIK cells) *vs*. <97.8 x 10^9 cells (HR, 0.98; *P*=0.87) and (B) in a subgroup of the immunotherapy group who received all scheduled 16 injections, ≥102 x 10^9 cells (median value of total injected CIK cells) *vs.* those who received < 102 x 10^9 cells (HR, 0.75; *P*=0.37). (C) among the whole immunotherapy group, ≥97.8 x 10^9 cells (median number of total injected CIK cells) *vs*. <97.8 x 10^9 cells (HR, 0.94; *P*=0.84) and (D) in a subgroup of the immunotherapy group who received all scheduled 16 injections, ≥102 x 10^9 cells (median value of total injected CIK cells) *vs.* those who received < 102 x 10^9 cells (HR, 0.80; *P*=0.81).

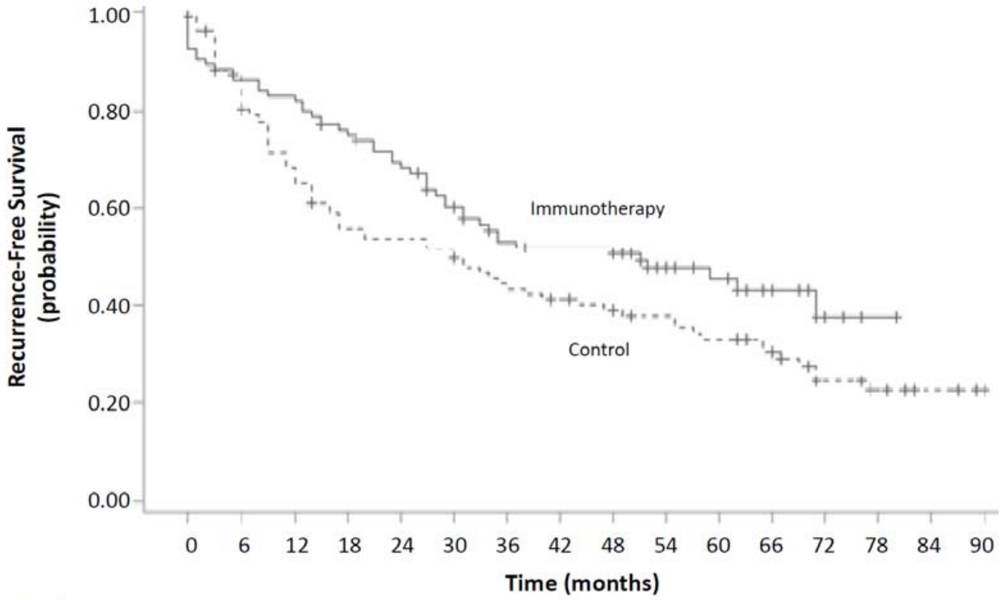

No. at risk

Immunotherapy	95	82	79	71	63	53	43	40	40	25	21	13	5	1		
Control	112	87	67	54	52	50	42	38	35	32	28	24	14	9	6	1

Supplementary Figure 4. Kaplan-Meier estimates of recurrence-free survival during off-treatment period: the immunotherapy group had lower risk of tumor recurrence (0.71; 95% CI, 0.49–1.01; *P*=0.03 by one-sided log-rank test). RFS was computed on all patients included in the efficacy population. Index dates were defined as the last date of CIK cell injection in the immunotherapy group and the date of randomization in the control group, respectively. Patients who had not progressed or died were censored on data cut-off.

Supplementary Table 1. Baseline demographics and disease characteristics of patients who did not undergo the extended follow-up

Variable	Immunotherapy (n=25)	Control group (n=39)	*P* value
Male sex, N (%)	18 (72.0)	29 (74.4)	0.83[e]
Age years, Mean (SE)	55.1 (6.9)	57.6 (11.2)	0.32[f]
Treatment modality, N (%)			0.64[g]
PEI	2 (8.0)	1 (2.6)	
RFA	18 (72.0)	23 (59.0)	
Surgical resection	5 (20.0)	15[d] (38.4)	
HCC stage, N (%)[a]			0.74[e]
Stage I	19 (76.0)	31 (79.5)	
Stage II	6 (24.0)	8 (20.5)	
Number of HCC, N (%)			0.42[e]
< 3	25 (100.0)	38 (97.4)	
≥ 3	0 (0.0)	1 (2.6)	
Size of HCC, cm			0.79[h]
Median (IQR)	2.1 (1.7–2.8)	2.0 (1.7–4.0)	
ECOG performance status, N (%)[b]			0.58[e]
0	17 (68.0)	29 (74.4)	
1	8 (32.0)	10 (25.6)	

Cause of liver disease, N (%)			0.84[g]
HBV infection only	21 (84.0)	28 (71.8)	
HCV infection only	2 (8.0)	6 (15.4)	
HBV+HCV co-infection	0 (0.0)	1 (2.6)	
Others	2 (8.0)	4 (10.2)	
Cirrhosis, N (%)[c]	18 (77.8)	27 (58.3)	0.81[e]
Biochemical analysis			
Alpha-fetoprotein, ng/mL			0.69[h]
Median (IQR)	4.2 (2.9–14.5)	6.0 (2.9–11.5)	
PIVKA-II, mAU/mL			0.48[h]
Median (IQR)	20.0 (14.0–24.0)	17.0 (14.0–22.0)	
Aspartate aminotransferase, IU/L			0.69[h]
Median (IQR)	35.0 (26.0–42.0)	36.0 (28.0–49.0)	
Alanine aminotransferase, IU/L			0.48[h]
Median (IQR)	28.0 (25.0–40.0)	33.0 (21.0–49.0)	
Alkaline Phosphatase, IU/L			0.30[h]
Median (IQR)	87.0 (77.0–132.0)	88.0 (68.0–101.0)	
Albumin, g/dL			1.00[h]
Median (IQR)	4.0 (3.8–4.2)	4.0 (3.9–4.2)	
Total bilirubin, mg/dL			0.79[h]
Median (IQR)	0.8 (0.7–1.2)	0.8 (0.6–1.1)	

Prothrombin time, sec			0.80[h]
Median (IQR)	13.6 (12.9–14.5)	13.9 (13.2–14.4)	
Creatinine, mg/dL			0.89[h]
Median (IQR)	0.9 (0.8–0.9)	0.9 (0.7–1.1)	
Platelet, $\times 10^3/mm^3$			0.04[h]
Median (IQR)	117 (78–158)	141 (118–167)	

NS, not significant; RFA, radiofrequency ablation; PEI, percutaneous ethanol injection; HCC, hepatocellular carcinoma; IQR, interquartile range; ECOG, Eastern Cooperative Oncology Group; HBV, hepatitis B virus; HCV, hepatitis C virus; PIVKA-II, protein induced by vitamin K absence-II.

Note. Data are expressed as n (%), mean (SE), or median (interquartile range [Q1–Q3]).

[a] The HCC staging was done according to AJCC staging system (6th edition).[46]

[b] The ECOG performance status assesses the daily living abilities of the patient, on a scale ranging from 0 (fully active) to 5 (dead).

[c] Liver cirrhosis was diagnosed by the presence of histological and radiological evidence.

[d] two of them underwent intrahepatic RFA in addition to surgical resection

[e] by chi-square test

[f] by two sample t-test

[g] Fisher's exact test

[h] by Wilcoxon rank sum test

Supplementary Table 2. Summary of imaging studies

	Immunotherapy (n = 114)	Control group (n = 112)	*P* value
Interval between each imaging study, days (per participants)			
Before 96 weeks, median (range)	84 (20–168)	83 (6–105)	0.74[a]
After 96 weeks, median (range)	119 (7–898)	119 (8–765)	0.82[a]
Number of overall imaging studies, N (per participants)			0.007[b]
Mean ± SD	12.9 ± 7.1	10.3 ± 7.3	
Number of each imaging modality, N (%) (overall participants)			0.11[c]
CT	1,391 (94.6%)	1,105 (95.9%)	
MRI	80 (5.4%)	47 (4.1%)	

SD, standard deviation; CT, computed tomography; MRI, magnetic resonance imaging.

[a] by Wilcoxon rank-sum test

[b] by two sample t-test

[c] by chi-square test

Supplementary Table 3. Summary of efficacy measures (efficacy population).

Outcome	Immunotherapy (n = 114)	Control (n = 112)	Hazard Ratio (95% CI)	*P* value
Recurrence-free survival rate				
24 months	72.5%	53.8%		0.002[a]
36 months	62.2%	43.3%		0.003[a]
48 months	49.2%	39.0%		0.07[a]
60 months	44.8%	33.1%		0.046[a]
72 months	41.0%	24.4%		0.008[a]
84 months	37.0%	22.4%		0.02[a]
Recurrence-free survival (mo)			0.67 (0.48–0.94)	0.010[b]
Median	46.0	30.0		
Overall survival rate				
24 months	100.0%	91.9%		
36 months	98.1%	88.7%		
48 months	97.1%	86.5%		
60 months	97.1%	85.3%		
72 months	91.9%	79.9%		
84 months	89.7%	76.1%		
Overall survival (mo)			0.33 (0.15–0.76)	0.006[b]
Median	NA	NA		

Cancer-specific survival rate				
24 months	100.0%	94.9%		
36 months	99.1%	91.6%		
48 months	98.1%	90.5%		
60 months	98.1%	89.2%		
72 months	94.5%	84.9%		
84 months	92.1%	80.9%		
Cancer-specific survival (mo)			0.33 (0.13–0.86)	0.017[b]
Median	NA	NA		

NOTE. Data are expressed as %, hazard ratio with 95% CI, or median.

Abbreviation: CI, confidence interval; NA, not applicable.

[a] by one-sided z-test

[b] by one-sided log-rank test

Supplementary Table 4. Univariate and multivariate analyses of factors associated with recurrence-free survival.

Variables	Univariate analysis		Multivariate analysis	
	HR (95% CI)	*P* value	HR (95% CI)	*P* value
Age (≥ 60 *vs.* < 60 years)	1.50 (1.07–2.12)	0.02	1.43 (1.01–2.03)	0.046
Sex (male *vs.* female)	1.32 (0.81–2.14)	0.26		
Etiology of liver disease (HBV or HCV *vs.* NBNC)	0.98 (0.57–1.71)	0.95		
Liver cirrhosis (yes *vs.* no)	1.20 (0.84–1.71)	0.32		
AFP (≥ 20 *vs.* < 20 ng/mL)	1.84 (1.17–2.89)	0.008	2.25 (1.41–3.60)	0.0007
Performance status (ECOG 0 *vs.* 1)	1.16 (0.80-1.68)	0.43		

Histological confirmation (yes *vs.* no)	0.79 (0.56–1.13)	0.20		
HCC maximal diameter (≥ 2 *vs.* < 2 cm)	1.17 (0.84–1.65)	0.35		
Treatment modality (RFA or PEI *vs.* surgery)	1.59 (1.08–2.35)	0.02	1.84 (1.22–2.77)	0.003
Platelet (≥ 140 *vs.* < 140 × 10^3/mm^3)	0.97 (0.69–1.37)	0.88		
AST (≥ 40 *vs.* < 40 IU/L)	1.24 (0.88–1.75)	0.23		
ALT (≥ 40 *vs.* < 40 IU/L)	0.93 (0.65–1.33)	0.69		
ALP (≥ 115 *vs.* < 115 IU/L)	1.10 (0.69–1.76)	0.68		
PIVKA-II (≥ 40 *vs.* < 40 AU/mL)	1.14 (0.56–2.34)	0.71		
Albumin (≥ 3.5 *vs.* < 3.5 g/dL)	0.94 (0.46–1.93)	0.87		
Bilirubin (≥ 1.2 *vs.* < 1.2 mg/dL)	1.12 (0.71–1.77)	0.63		

Prothrombin time (≥ 13 *vs.* < 13 sec)	1.05 (0.67–1.64)	0.84		
Creatinine (≥ 1.2 *vs.* < 1.2 mg/dL)	1.39 (0.73–2.65)	0.31		
Treatment group (immunotherapy *vs.* control group)	0.67 (0.48–0.94)	0.01[a]	0.69 (0.49–0.97)	0.016[a]

Abbreviation: HR, hazard ratio; CI, confidence interval; HBV, hepatitis B virus; HCV, hepatitis C virus; NBNC, non-HBV and non-HCV; AFP, alpha-fetoprotein; ECOG, Eastern Cooperative Oncology Group; HCC, hepatocellular carcinoma; RFA, radiofrequency ablation; PEI. Percutaneous ethanol injection; AST, aspartate aminotransferase; ALT, alanine aminotransferase; ALP, alkaline phosphatase; PIVKA-II, protein induced by vitamin K absence-II.

[a] by one-sided test. Otherwise, two-sided test was used.

Supplementary Table 5. Post-recurrence treatment modalities in the immunotherapy group and the control group.

Treatment modalities	Immunotherapy (n = 63)	Control (n = 68)
Transarterial chemoembolization	182	219
Radiofrequency ablation	52	53
Percutaneous ethanol injection	50	42
Surgical resection	9	15
Liver transplantation	11	4
Sorafenib	10	7
Conventional chemotherapy	16	9
Radiation therapy	17	21
Proton therapy	0	1
**Total**	347	371

Supplementary Table 6. Change in serum level of alpha-fetoprotein (efficacy population).

Variable	Immunotherapy (n = 114)	Control (n = 112)	*P* value[a]
Alpha-fetoprotein (ng/mL)			
Baseline	11.3 ± 18.6	9.2 ± 13.6	
End of study	16.1 ± 40.5	54.0 ± 439.3	
Change from baseline	5.3 ± 42.0	50.3 ± 463.2	0.57
P value[b]	0.57	0.75	

NOTE: Data are expressed as mean ± SD.

[a] Wilcoxon rank-sum test comparing changes in indicated parameters of two groups from baseline.

[b] Wilcoxon signed-rank test comparing changes in indicated parameters within each group from baseline.
